# 3D Simulation Study for a Pneumatic Nozzle–Cylindrical Flapper System

**DOI:** 10.3390/s26092578

**Published:** 2026-04-22

**Authors:** Peimin Xu, Kazuaki Inaba, Toshiharu Kagawa

**Affiliations:** 1 Department of Transdisciplinary Science and Engineering, Institute of Science Tokyo, Tokyo 152-8550, Japan; xu.p.aa@m.titech.ac.jp; 2 Institute of Science Tokyo, Tokyo 152-8550, Japan; tkagawa0256@gmail.com

**Keywords:** high-speed rotation spindle with aerostatic bearings, nozzle flapper, pneumatic, machining

## Abstract

With the increasing demand for higher efficiency in semiconductor machining, air spindles with compensation systems have attracted growing attention. The pneumatic nozzle–cylindrical flapper is a promising sensing approach due to its high precision and suitability for displacement measurement of high-speed rotating bodies. However, its complex three-dimensional flow behavior leads to significant deviations from conventional nozzle–flat flapper models, limiting its practical application. This study aims to clarify the flow mechanisms governing the nozzle–cylindrical flapper system and to establish a reliable framework for predicting its static characteristics. A computational fluid dynamics model is developed to analyze gas flow within the micron-scale clearance under varying gap sizes and angular orientations, and the results are validated against experimental measurements. The analysis shows that curvature plays a dominant role in the flow behavior. Increasing curvature enhances inertia-driven acceleration and weakens viscous effects while simultaneously inducing strong recirculation due to flow wrapping around the cylindrical surface. These competing mechanisms explain the observed deviations from conventional models and cannot be captured by two-dimensional approaches. Based on the numerical results, a mass flow rate compensation coefficient is introduced and correlated with the momentum compensation coefficient. A quadratic relationship between the two coefficients is identified, indicating a common recirculation-driven mechanism. These findings support previous semi-empirical assumptions and provide a basis for predicting static characteristics with reduced reliance on experimental calibration.

## 1. Introduction

With the rapidly increasing global demand for the development of emerging technologies such as artificial intelligence (AI) and big data, semiconductors, as one of the core hardware foundations supporting these technologies, have experienced supply shortages that have exerted significant impacts on worldwide technological advancement and economic development [1,2] Air spindles, characterized by non-contact support, offer advantages such as low friction and long service life. By enabling higher rotational speeds and longer continuous machining durations, air spindles present considerable potential for improving the efficiency of semiconductor manufacturing processes [3]. However, due to their susceptibility to external disturbances [4], combined with the fact that non-contact support structures are prone to generating gyroscopic effects that can induce positional deviations during operation [5], the machining accuracy of air spindles may be adversely affected. Motivated by this concern, a compensation system based on the nozzle–flapper displacement sensing principle was proposed. Through collaborative research, both the existence of gyroscopic effects during air spindle machining and the effectiveness of the proposed compensation system in mitigating such effects were experimentally verified [6]. Although, in the development of the aforementioned compensation system, the feasibility of employing a nozzle–cylindrical flapper as a displacement sensor for the first time to measure a rotating body at 20,000 rpm was demonstrated [7], it is important to emphasize that the performance of such sensing systems is strongly governed by the underlying flow behavior within the nozzle–flapper clearance. In particular, the pressure–clearance relationship, which defines the static characteristics of the sensor, directly governs the sensitivity, linearity, and stability of the displacement measurement. However, in practical applications, the measured surface is inherently cylindrical rather than planar, and thus the nozzle–cylindrical flapper represents the actual sensing configuration. Previous studies have also shown that its static characteristics differ significantly from those of the conventional nozzle–flat flapper system [8], indicating that existing models cannot be directly applied to evaluate sensor performance in such configurations. For the traditional nozzle–flat flapper system, which has a long history of development, on the one hand, highly accurate empirical formulations [9,10] as well as purely theoretical calculation models [11,12] have been established. The static characteristics of the nozzle–flat flapper system are governed by the supply pressure and its structural parameters and demonstrate high repeatability. This inherent consistency enables standardized design and manufacturing practices and has led to the accumulation of extensive and highly reliable experimental data [13,14].

From a fluid dynamics perspective, the interaction between a jet and a curved surface is not a new problem and has been extensively studied in classical aerodynamics, particularly in the context of the Coandă effect, which describes the tendency of a jet to attach to a nearby curved surface due to entrainment and pressure gradients [15]. Experimental and theoretical studies have shown that jet attachment to convex surfaces, such as cylindrical geometries, significantly affects pressure distribution and flow stability [16]. In addition, numerical investigations have demonstrated that curvature influences jet deflection, boundary layer development, and recirculation behavior [17]. However, most existing studies focus on macroscopic scales and simplified geometries, and their conclusions cannot be directly extended to the present problem [18]. Therefore, while the fundamental flow phenomena such as jet attachment and recirculation have been extensively studied, their behavior under micron-scale confined conditions in nozzle–cylindrical flapper systems remains insufficiently understood, particularly in relation to sensor performance. In contrast, for the nozzle–cylindrical flapper system, the feasibility of its application has only been verified in recent years, and the currently available experimental data are relatively limited, rendering them insufficient for extensive use in systematic industrial design. Moreover, the present configuration involves micron-scale clearances and geometric asymmetry, which lead to complex flow features such as coupled inertia–viscosity transitions and localized recirculation. These characteristics differ fundamentally from those considered in classical jet–surface interaction studies. Consequently, the semi-empirical formulations and theoretical models developed for nozzle–flat flapper systems cannot be directly applied to the static characteristic calculations of nozzle–cylindrical flapper systems. Although our previous study proposed a semi-empirical theoretical model capable of achieving high-accuracy predictions of static characteristics with only a small amount of experimental data [8], the development of nozzle–cylindrical flapper systems still inevitably relies on costly physical experiments with stringent accuracy requirements. To reduce the development cost of cylindrical nozzle–flapper systems and to obtain more detailed flow-field information as well as direct observations of the internal flow behavior, computational fluid dynamics (CFD) simulation is of great importance as an effective and flexible analysis tool. Although two-dimensional numerical simulations of nozzle–flapper configurations have been reported [12], such approaches are inherently limited to axisymmetric or geometrically simplified models. For non-axisymmetric configurations, such as the cylindrical nozzle–flapper considered in this study, a two-dimensional framework cannot accurately reproduce the three-dimensional geometry or the associated flow phenomena, including asymmetric recirculation and curvature-induced effects. On the other hand, while extensive three-dimensional CFD studies on hydraulic nozzle–flapper systems are available in the literature [19,20,21], these investigations are primarily based on incompressible liquid flows and therefore do not capture the compressibility effects that are essential in pneumatic systems. To the authors’ best knowledge, no prior studies have addressed pneumatic cylindrical nozzle–flapper configurations, which require fully three-dimensional CFD analysis to simultaneously account for non-axisymmetric geometry and compressible flow physics. Therefore, the objective of this study is to clarify the flow mechanisms governing the nozzle–cylindrical flapper system and to establish a physically grounded framework for predicting its static characteristics with direct relevance to sensing performance. A three-dimensional computational fluid dynamics simulation framework is developed to investigate the flow behavior within the clearance region. The significance and necessity of this approach can be summarized as follows: The flow characteristics of air within the clearance region between the nozzle and the flapper are investigated, providing fundamental insights into the underlying fluid dynamic behavior. Due to the micron-scale irregular geometric features of the nozzle–cylindrical flapper, conventional experimental flow visualization techniques are difficult to apply. In contrast, CFD simulations enable clear and detailed observation of the flow field within the clearance region. Compared with physical experiments that involve high costs and stringent precision requirements, CFD simulations offer advantages in terms of lower cost, higher numerical accuracy, and significantly shorter development cycles. Owing to the geometric non-rotational symmetry of the nozzle–cylindrical flapper configuration, two-dimensional CFD simulations are incapable of capturing the true flow characteristics. Therefore, a three-dimensional CFD approach is essential to accurately resolve the flow behavior.

The remainder of this paper is organized as follows. Section 2 further examines the differences in discharge characteristics between the nozzle–cylindrical flapper and the conventional nozzle–flat flapper and discusses the limitations and underlying assumptions of the previously proposed semi-empirical theoretical model for the nozzle–cylindrical flapper system. Section 3 describes the detailed configurations of the numerical simulations and experimental setups, including the governing equations and the selection of turbulence models employed in the CFD analysis. Section 4 presents and discusses the simulation and experimental results, mesh independence tests are conducted, and the numerical results are validated through physical experiments. CFD simulations are employed to elucidate the airflow characteristics within the clearance region between the nozzle and the flapper, thereby enabling the calibration of the previously established semi-empirical theoretical model. Finally, Section 5 summarizes the main findings and conclusions of this study.

## 2. Discharge Characteristic Analysis of the Nozzle-Cylindrical Flapper System

**Figure 1:**
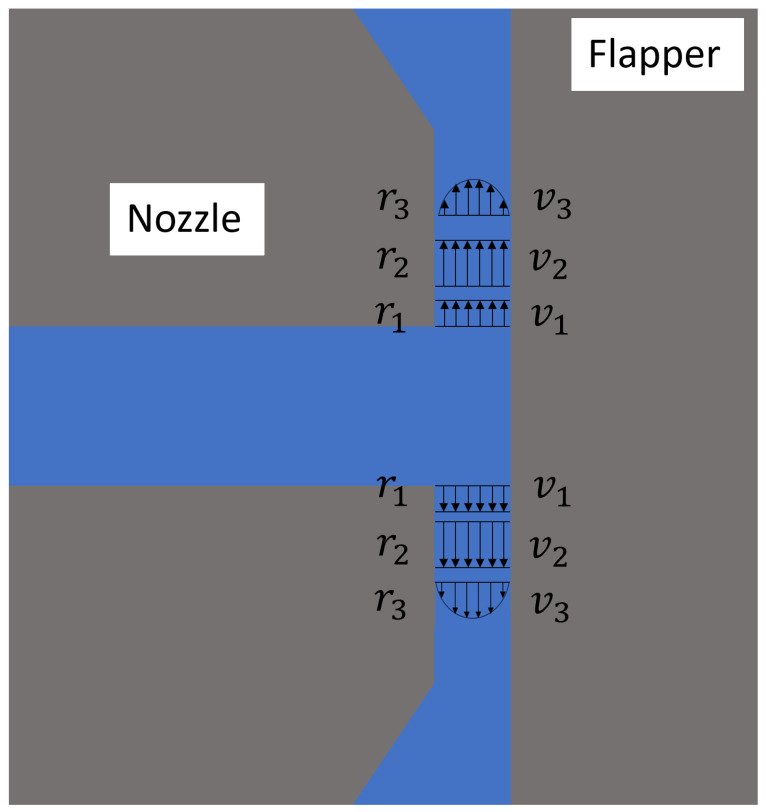
Geometric plane parallel to the axis of the cylindrical flapper (pure flat case) [8].

In previous studies, the differences in discharge flow characteristics between the nozzle–cylindrical flapper and the conventional nozzle–flat flapper were investigated. As illustrated in Figure 1, the most characteristic geometric and flow feature of the traditional nozzle–flat flapper system is that both the nozzle outlet and the flapper surface are planar. After being discharged from the nozzle, the airflow undergoes two distinct stages: an initial acceleration stage driven by pressure-induced kinetic energy, followed by a viscosity-dominated stage in which boundary layers progressively develop owing to the constant clearance between the nozzle and the flapper walls.

Although geometric scaling differences exist between the nozzle–flapper configuration and an air thrust bearing, their discharge characteristics exhibit notable similarities. Consistent with previous conclusions, in the nozzle–flat flapper system, the fixed clearance between the nozzle and the flapper promotes the formation of a viscosity-dominated flow regime, resulting in substantial viscous resistance and consequently reducing the discharge mass flow rate.

**Figure 2:**
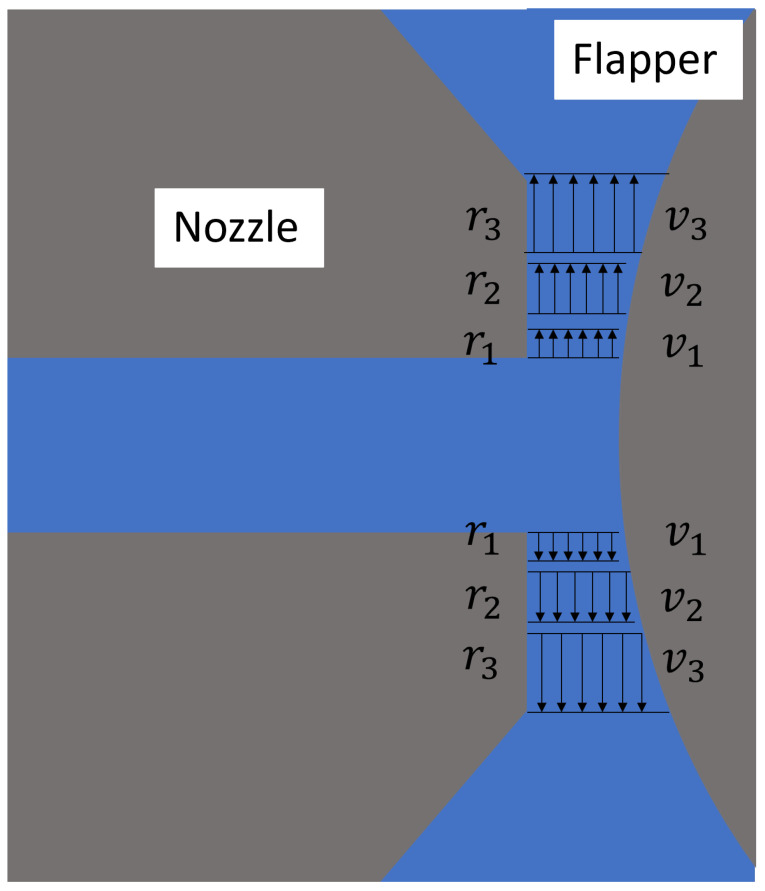
Geometric plane perpendicular to the axis of the cylindrical flapper (pure arc case) [8].

In contrast, for the nozzle–cylindrical flapper system, although high accuracy has been achieved in near-nozzle flow analysis and modeling, the influence of additional factors becomes non-negligible when the nozzle lip—defined as the axial length from the nozzle orifice to the nozzle edge—is taken into consideration. As shown in Figure 2, previous analyses assumed that the compressed air discharged from the nozzle completely fills the entire clearance region between the nozzle, the lip, and the flapper. However, pressure distribution data obtained from nozzle–flapper measurements [22] indicate that the static pressure of the airflow has already decreased to atmospheric pressure before reaching the nozzle lip edge. This observation leads to the conclusion that the effective discharge area of the nozzle–cylindrical flapper is smaller than the geometric outlet area of the nozzle when the lip is included.

Furthermore, when combined with flow separation effects and the entrainment effect induced by the high-speed jet, it can be inferred that, for the cylindrical flapper, regions with relatively large curvature—where flow separation is more pronounced—exhibit significant backflow and enhanced turbulent dissipation near the outermost edge of the nozzle lip due to entrainment. Based on the above reasoning, it follows that, for the nozzle–cylindrical flapper, flow cross-sections at different angular positions (corresponding to different clearance growth rates between the nozzle and the flapper) are subjected to two distinct velocity-reducing mechanisms: viscous resistance and turbulent dissipation. While viscous resistance is the dominant factor in the nozzle–flat flapper system, excessive curvature in the nozzle–cylindrical flapper system gives rise to backflow-induced turbulent dissipation.

Consequently, for the nozzle–cylindrical flapper, regions with moderate curvature are expected to exhibit the highest flow velocities, as both viscous resistance and turbulent dissipation are minimized in these regions.

## 3. Experimental Preparation and Simulation Methods

### 3.1. Experimental Design

**Figure 3:**
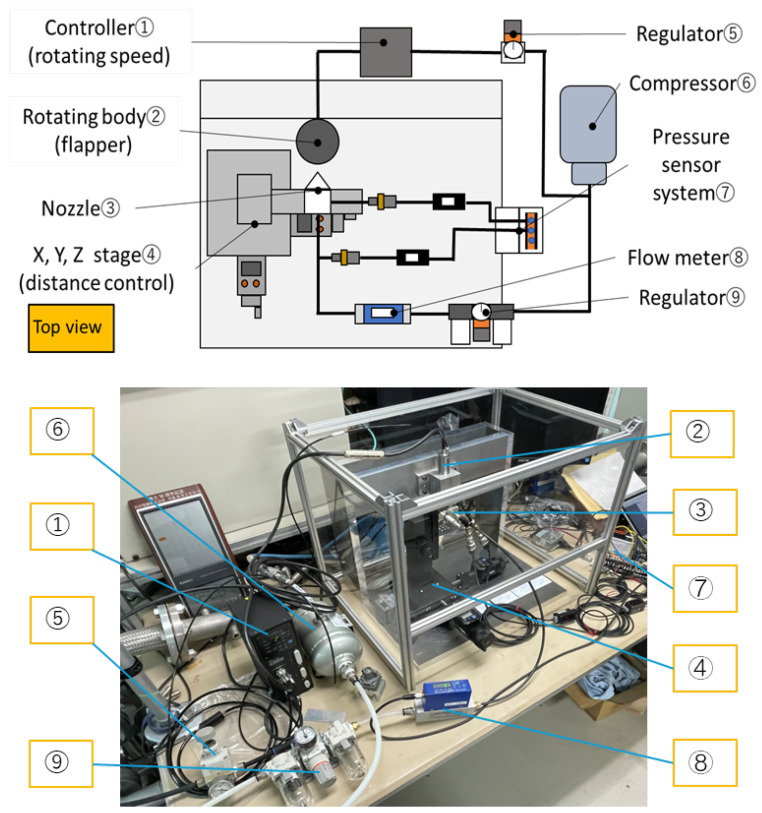
Experimental system for the static characteristic measurement of nozzle–cylindrical flapper system.

To verify the reliability of the numerical simulation results, an experimental system was designed and constructed to measure the clearance between the nozzle and the cylindrical flapper as well as the corresponding control pressure. As illustrated in Figure 3, a physical prototype of the experimental setup was assembled according to the experimental schematic. The system consists of a rotational speed controller, a rotating cylindrical flapper, a nozzle assembly (all from industria Co., Ltd., Iruma, Saitama, Japan), an XYZ micrometer positioning stage (CHUO PRECISION INDUSTRIAL CO., LTD., Tokyo, Japan), a pressure regulator (SMC Corporation, Tokyo, Japan), an air compressor (MAX Co., Ltd., Tokyo, Japan), a pressure sensing system (KEYENCE Corporation, Osaka, Japan), and a flow meter (TOKYO METER CO., LTD., Kawasaki, Kanagawa, Japan).

The cylindrical flapper has a diameter of 16 mm, and its rotational speed is controlled at 20,000 rpm using the speed controller. The nozzle has a diameter of 0.8 mm and is equipped with an orifice of 0.4 mm in diameter. An XYZ micrometer positioning stage is employed to ensure that the nozzle axis is perpendicular to the axis of the cylindrical flapper and to precisely adjust the clearance between the nozzle and the flapper within a range of 0–30 μm.

Compressed air is supplied by the air compressor with a maximum pressure capacity of 4 MPa, while the supply pressure to the nozzle is regulated to 500 and 600 kPaG using a pressure regulator. A pressure-sensing system is used to measure and record the variation of the control pressure as a function of the clearance between the nozzle and the cylindrical flapper.

### 3.2. Rotation Effect on Static Characteristic of Nozzle–Cylindrical Flapper

**Figure 4:**
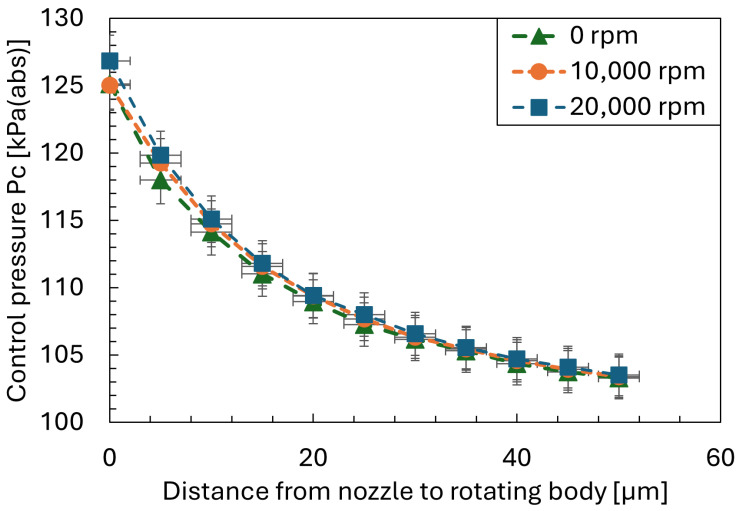
Experimental Comparison of Static Characteristics under Different Rotational Speeds.

Although previous studies have indicated that the effect of rotational speed on the nozzle–flapper system can be neglected at speeds up to 20,000 rpm, additional experimental validation was conducted for completeness. The experiments were performed under a supply pressure of 511 kPaG, with a nozzle diameter of 1.6 mm and an orifice diameter of 0.2 mm. Static characteristics were measured at rotational speeds of 0, 10,000 rpm, and 20,000 rpm. As shown in Figure 4, the results demonstrate that there is no significant difference in the static characteristics of the nozzle–cylindrical flapper system among the three rotational conditions. Based on this observation, the CFD simulations in the present study were conducted under non-rotating conditions.

The repeatability of the experimental measurements was evaluated over three independent trials. The measured displacement exhibited a repeatability error of less than 6.7%, while the control pressure showed a repeatability error of less than 7.2% [8].

The drift was evaluated under constant operating conditions at 20,000 rpm. The variation of the output signal over a duration of 10 min was found to be within ±1% of full scale, which is comparable to the measurement uncertainty. Therefore, the drift can be considered negligible for the present study.

### 3.3. Governing Equations

The flow field inside the nozzle–flapper configuration is characterized by turbulent motion. Although direct numerical simulation (DNS) of the Navier–Stokes (N–S) equations is capable of resolving all turbulence scales, such an approach is computationally prohibitive due to its excessive requirements in computational time and memory resources. In practical engineering applications, the primary interest lies in the prediction of time-averaged quantities, including the mean velocity field, mean scalar variables, and turbulence-related forces.

Accordingly, a statistical averaging procedure is applied to the N–S equations, resulting in the Reynolds-averaged Navier–Stokes (RANS) formulation, which can be expressed as follows [23]:

(1) $$\frac{\partial \rho }{\partial t}+\frac{\partial }{\partial x_{i}}\left(\rho u_{i}\right)=0,$$

(2) $$\frac{\partial }{\partial t}\left(\rho u_{i}\right)+\frac{\partial }{\partial x_{j}}\left(\rho u_{i}u_{j}\right)=-\frac{\partial p}{\partial x_{i}}+\frac{\partial \sigma_{ij}}{\partial x_{j}}-\frac{\partial }{\partial x_{j}}\left(\rho u_{i}u_{j}^{\prime }\right).$$

Here, $u_{i}$ denotes the Reynolds-averaged velocity component, ρ represents the fluid density, *p* is the static pressure, $u_{i}^{\prime }$ is the fluctuating velocity component, and $\sigma_{ij}$ denotes the viscous stress tensor.

Compared with the instantaneous N–S equations, the RANS formulation introduces the Reynolds stress term, which accounts for the momentum transport induced by turbulent fluctuations. The presence of this additional term leads to a closure problem, as the number of unknowns exceeds the number of governing equations. To close the system and ensure mathematical solvability, an appropriate turbulence model must be employed to approximate the effects of unresolved turbulent motions on the mean flow field, without explicitly resolving the full range of turbulence scales.

### 3.4. Turbulence Models

Although the RNG *k*–ε turbulence model improves the prediction capability of the standard *k*–ε formulation by incorporating strain-rate effects and swirl corrections, its applicability remains limited for flows dominated by strong near-wall effects, adverse pressure gradients, and flow separation. In the nozzle–cylindrical flapper system considered in this study, the discharge flow within the micron-scale clearance region is highly sensitive to wall proximity, curvature-induced separation, and entrainment-induced backflow near the nozzle lip. Under such conditions, turbulence models relying on wall functions, including the RNG *k*–ε model, may lead to inaccurate predictions of velocity gradients and turbulent dissipation.

To address these limitations, the shear stress transport (SST) *k*–ω turbulence model is adopted. The SST model combines the advantages of the *k*–ω formulation in the near-wall region with those of the *k*–ε model in the free-stream region through blending functions, enabling accurate resolution of boundary layer development and flow separation without the use of empirical wall functions.

The transport equations for the turbulent kinetic energy *k* and the specific dissipation rate ω in the SST *k*–ω model are given by [24]:

(3) $$\frac{\partial (\rho k)}{\partial t}+\frac{\partial (\rho u_{j}k)}{\partial x_{j}}=P_{k}-\beta^{*}\rho k\omega +\frac{\partial }{\partial x_{j}}\left[\left(\mu +\sigma_{k}\mu_{t}\right)\frac{\partial k}{\partial x_{j}}\right],$$

(4) $$\frac{\partial (\rho \omega )}{\partial t}+\frac{\partial (\rho u_{j}\omega )}{\partial x_{j}}=\alpha \frac{\omega }{k}P_{k}-\beta \rho \omega^{2}+\frac{\partial }{\partial x_{j}}\left[\left(\mu +\sigma_{\omega }\mu_{t}\right)\frac{\partial \omega }{\partial x_{j}}\right]+2\left(1-F_{1}\right)\rho \sigma_{\omega 2}\frac{1}{\omega }\frac{\partial k}{\partial x_{j}}\frac{\partial \omega }{\partial x_{j}},$$

where $P_{k}$ denotes the production term of turbulent kinetic energy, $\mu_{t}$ is the turbulent eddy viscosity, and $F_{1}$ is the blending function controlling the transition between the near-wall *k*–ω model and the outer-region *k*–ε behavior.

The turbulent eddy viscosity is defined as:

(5) $$\mu_{t}=\frac{\rho a_{1}k}{\max(a_{1}\omega ,SF_{2})},$$

where *S* is the invariant measure of the strain rate tensor, $a_{1}$ is a model constant, and $F_{2}$ is the second blending function.

The introduction of the eddy-viscosity limiter prevents excessive turbulence production and enables more accurate prediction of flow separation under adverse pressure gradients. This feature is particularly important for the present nozzle–cylindrical flapper system, where local flow separation, backflow, and entrainment-induced turbulent dissipation near the nozzle lip play a dominant role in determining the effective discharge characteristics. Therefore, compared with the RNG *k*–ε model, the SST *k*–ω model is more suitable for resolving the complex near-wall flow phenomena inherent in the nozzle–cylindrical flapper configuration.

### 3.5. Grid Generation and Grid Independence Verification

Prior to conducting a systematic CFD analysis of the nozzle–cylindrical flapper system, a mesh independence study was performed to ensure the numerical reliability of the simulations. Based on the design parameters and operating conditions listed in Table 1, the computational domains of the nozzle–cylindrical flapper configuration were established. According to previous studies, the influence of rotational speed on the static characteristics of the nozzle–cylindrical flapper system can be neglected when the rotational speed is below 20,000 rpm [8]. Therefore, the present simulations are conducted under a stationary flapper condition. The walls are assumed to be stationary with no-slip boundary conditions. Since thermal effects are neglected, the walls are treated as adiabatic. The simulations are performed using ANSYS FLUENT 2024 R1 with a pressure-based solver, and the energy equation is solved to account for potential compressibility effects. The fluid viscosity is modeled using Sutherland’s law. The pressure–velocity coupling is handled using the coupled algorithm. Convergence is assessed based on both residuals and physical quantities, with residuals of continuity and momentum reduced below $10^{-3}$ and turbulence quantities below $10^{-6}$, while key variables such as mass flow rate remain stable within 1%. The SST *k*–ω turbulence model is employed, and both curvature correction and production limiter options are activated.

**Figure 5:**
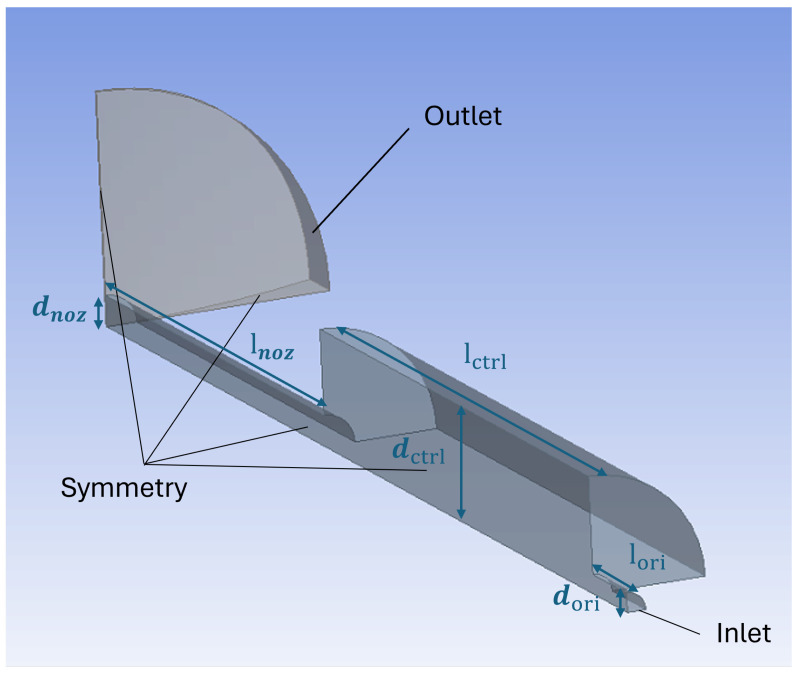
Three-dimensional geometric model of the nozzle–cylindrical flapper system.

Although the nozzle–cylindrical flapper geometry is not rotationally symmetric, it exhibits two planes of symmetry parallel and perpendicular to the cylindrical flapper axis. To reduce computational cost while maintaining sufficient accuracy, a three-dimensional quarter-domain (1/4) model, as shown as Figure 5, was constructed according to the parameters in Table 1.

**Figure 6:**
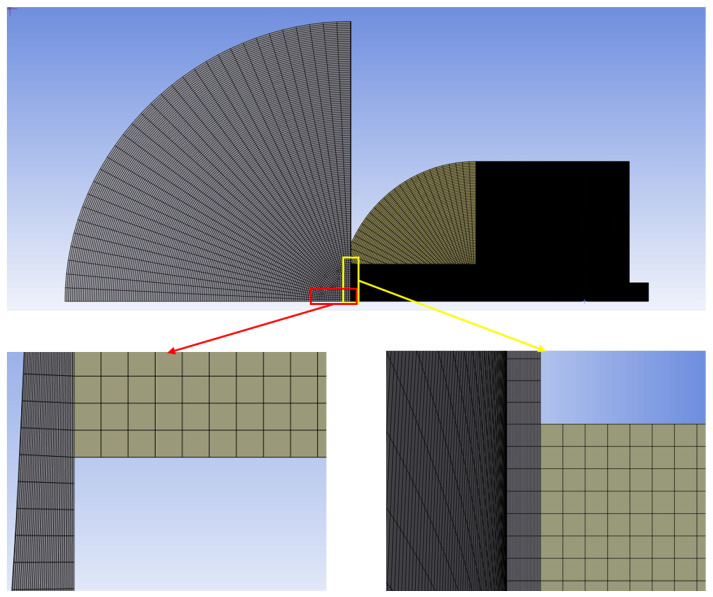
Grid systems used for numerical simulations.

The mesh generation was carried out using the ANSYS Meshing 2024 R1 module, and the resulting grid structure is illustrated in Figure 6.

To rigorously assess mesh independence, the case with the minimum nozzle–flapper clearance of x = 30 μm was selected as the benchmark condition. The mass flow rate, which is the primary quantity of interest in this study, was adopted as the evaluation metric. Under identical operating conditions, seven different mesh refinement schemes were examined, as summarized in Table 2, together with their corresponding mass flow rates.

**Figure 7:**
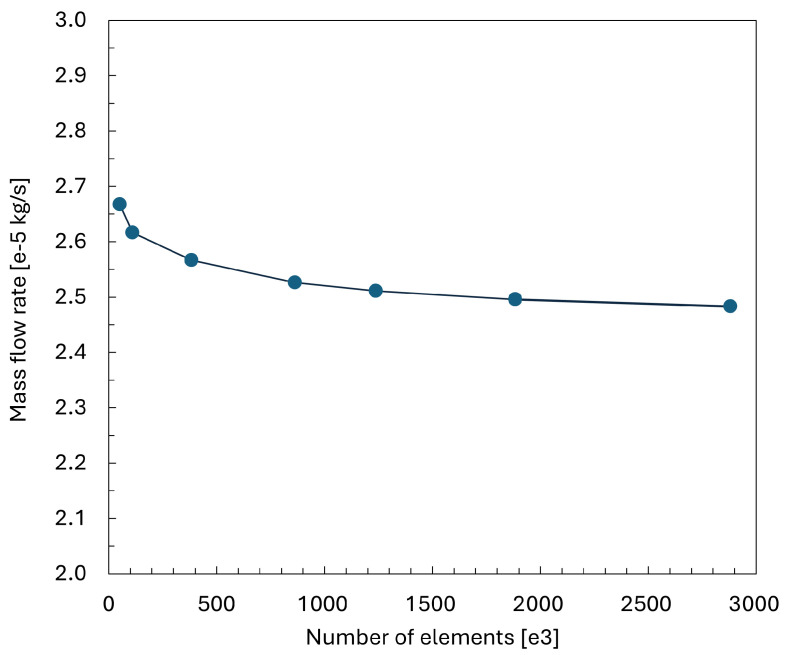
Mesh independence study results.

As shown in Figure 7, although the mass flow rate continues to decrease with increasing mesh resolution, the rate of reduction is significantly attenuated from Mesh ID 4 to Mesh ID 7. A further estimation indicates that, within the clearance range of 30–35 μm for the cylindrical flapper nozzle, the differential of the mass flow rate with respect to unit micrometer is approximately 0.05 kg/(s·μm), whereas the variation around Mesh ID 5 is only 0.015 kg/(s·μm). Considering both computational accuracy and computational cost, Mesh ID 5 was therefore selected for all subsequent simulations. Specifically, Mesh ID 5 employs a structured boundary layer mesh near the wall with approximately 30 prism layers and a growth rate of 1.15. The mesh is further refined in critical regions, such as the nozzle exit and the gap, to accurately capture strong flow gradients. The mesh quality is evaluated with a maximum skewness of 0.544 (average 0.04) and a minimum orthogonal quality of 0.086 (average 0.987), indicating overall good mesh quality despite a small number of locally low-quality cells. The near-wall resolution is designed to achieve low $y^{+}$ values (average ≈1.85), indicating that the viscous sublayer is largely resolved, corresponding to a low-Reynolds-number treatment rather than wall functions.

## 4. Results and Discussion

### 4.1. Validation of Simulation

After completing the preparatory work for both the CFD simulations and the physical experiments described in the preceding sections, simulation data and experimental data were obtained synchronously under identical operating conditions defined for each case. For the nozzle–cylindrical flapper system, the most critical performance metric is its static characteristic, namely the relationship between the control pressure and the clearance between the nozzle and the cylindrical flapper. Therefore, this static characteristic is adopted as the primary criterion for validating the numerical simulations.

It is worth noting that, for the case with a simulated clearance of x=30μm, the corresponding control pressure is close to the control pressure measured in the physical experiment at a nominal clearance of x=0μm. This discrepancy can be explained as follows. In the physical experiments, due to the high-speed rotation of the cylindrical flapper as well as high-frequency vibrations induced by the motor, the nozzle–cylindrical flapper system cannot achieve true mechanical contact at the nominal zero-clearance position. Consequently, a deviation exists between the experimental zero-clearance reference and the ideal zero-clearance condition assumed in the numerical simulations.

However, with respect to the static characteristics of the nozzle–cylindrical flapper system, the experiments were conducted at a rotational speed of 20,000 rpm. The measurement uncertainty of the displacement sensor (LD-243-C7) is specified as ±1μm, while the pressure sensor (AP-13S) has a full-scale measurement uncertainty of ±0.5% F.S. The repeatability of the experimental measurements was evaluated over three independent trials. The measured displacement exhibited a repeatability error of less than 6.7%, while the control pressure showed a repeatability error of less than 7.2% [8].

In the experiment, the nominal clearance of 0μm is defined as the condition at which the nozzle physically contacts the high-speed rotating surface. However, due to the surface machining roughness of the rotating body and misalignment tolerance (approximately ±27μm), the exact determination of the zero-clearance position cannot be as precise as in the CFD simulations, where an ideal smooth surface is assumed. Therefore, a certain level of uncertainty is inherently associated with the experimental reference point.

Nevertheless, as demonstrated by the overall agreement between simulation and experimental results in terms of both magnitude and trend, and considering that the absolute value of the control pressure at a specific clearance is of secondary importance, the key factor lies in the pressure differential, that is, the variation of control pressure as a function of the clearance. Based on this consideration, the present study aligns the simulated case with a clearance of 30μm with the experimental case at a nominal clearance of 0μm as a common reference point. Subsequently, the simulated cases with clearances of 45μm and 60μm are compared with the experimental results obtained at nominal clearances of 15μm and 30μm, respectively, to perform an effective validation of the simulation results.

This approach enables a more detailed investigation of the flow characteristics and provides a meaningful comparison with the previously developed theoretical model [8], thereby yielding a more realistic interpretation of the underlying physical phenomena.

**Figure 8:**
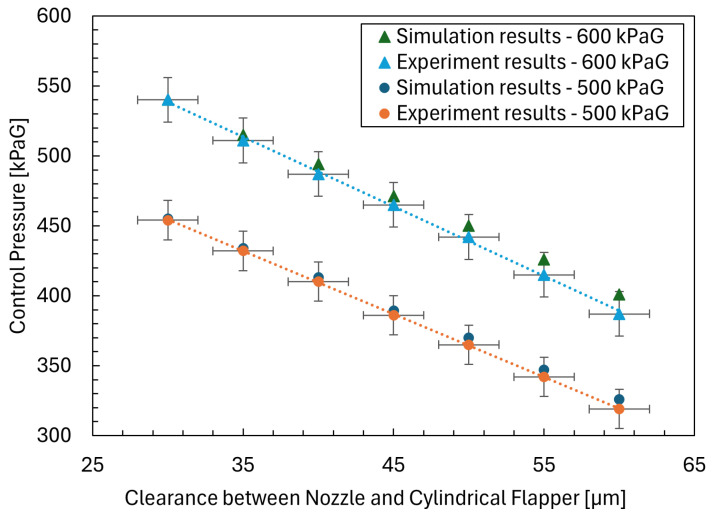
Validation results of simulation.

Based on the results shown in Figure 8, the credibility of the numerical simulations can be validated according to the following observations.

First, although a discrepancy exists between the numerical simulations and the physical experiments in terms of the clearance corresponding to the same initial control pressure, once a common reference point is defined, the differences in the control pressure obtained from the simulations and the experiments under identical clearance variations remain within the systematic error range of the experimental setup.

Second, by evaluating the pressure differential of the control pressure with respect to the clearance, the deviations between the numerical and experimental results are also found to be within the experimental system error. Moreover, both the numerical simulations and the physical experiments exhibit a clear and consistent linear relationship between the control pressure and the nozzle-to-flapper clearance.

From the perspective of sensor performance analysis, the supply pressure was set to 500 kPaG, with a flapper diameter of 16 mm, a nozzle orifice diameter of 0.8 mm, and an orifice diameter of 0.4 mm.

The sensitivity obtained from the numerical simulation for the relationship between the nozzle–cylindrical flapper clearance and the control pressure is 4.3kPa/μm, whereas the experimentally measured sensitivity is 4.5kPa/μm. Both the simulation and experimental results demonstrate a linear relationship between the control pressure and the nozzle-to-flapper clearance within a range of 30μm. The linearity was quantitatively evaluated using the maximum deviation from the least-squares fitted line normalized by the full-scale output. For the supply pressure of 500 kPaG, the linearity error was approximately 2.7% FS, while for 600 kPaG, it was approximately 2.9% FS. In both cases, the R^2^ value exceeded 0.998, indicating excellent linearity.

These results demonstrate that the proposed system maintains stable linear characteristics across different supply pressures.

Taken together, these results demonstrate that the numerical simulations accurately capture the static characteristics of the nozzle–cylindrical flapper system, thereby validating the effectiveness and reliability of the proposed simulation methodology.

### 4.2. Numerical Analysis of Nozzle–Cylindrical Flapper System

After completing the validation of the numerical simulations, the flow characteristics of the nozzle–cylindrical flapper system are investigated in detail. As discussed in Section 2, the nozzle–cylindrical flapper configuration involves an irregular clearance geometry and micrometer-scale dimensions, which make conventional experimental flow visualization techniques extremely difficult to apply in the present study. Under such circumstances, velocity contour distributions obtained from CFD simulations play a crucial role in revealing the internal flow behavior of the system.

In this chapter, the discharge characteristics of the nozzle–cylindrical flapper system are first clarified. The differences and similarities between a purely arc-shaped plane and a purely flat plane under identical clearance conditions are examined, with the aim of verifying the flow hypotheses proposed in Section 2 as well as in our previous studies.

After completing simulations under the aforementioned conditions for nozzle–cylindrical flapper clearances of 30, 35, 40, 45, 50, 55, and 60 μm, streamline visualizations were obtained for all cases. In addition, the distributions of velocity, pressure, and density were extracted for each case on planes oriented at $0^{\circ }$ (pure flat plane), $15^{\circ }$, $30^{\circ }$, $45^{\circ }$, $60^{\circ }$, $75^{\circ }$, and $90^{\circ }$ (pure arc plane) with respect to the axis of the cylindrical flapper.

**Figure 9:**
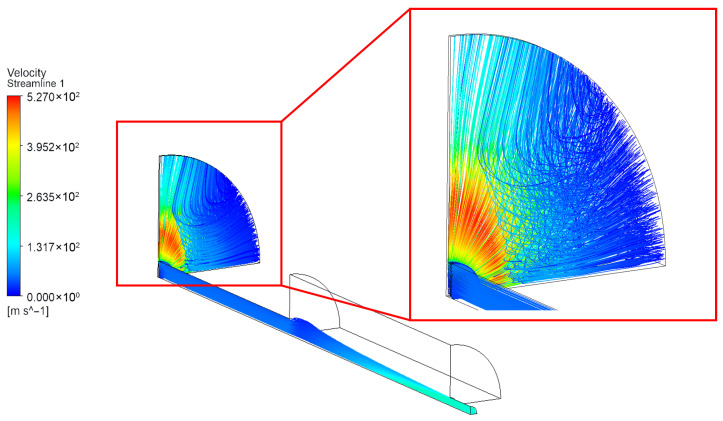
The stream line of nozzle–cylindrical flapper’s clearance = 45 μm.

Among all simulated cases, the clearance of 45 μm exhibited the most representative overall flow characteristics. Therefore, the corresponding streamline distribution, shown in Figure 9, is selected to provide a preliminary analysis of the global flow features in the nozzle–cylindrical flapper system.

As shown in Figure 9, the streamline distribution clearly reflects the previously discussed partial characteristics of the nozzle–cylindrical flapper system. Specifically, three representative planes can be identified, namely the pure flat plane, the mixed-arc plane, and the pure arc plane. Owing to the lower discharge resistance associated with the pure arc plane, the airflow exhibits a tendency to migrate from planes with lower curvature toward those with higher curvature.

Furthermore, the pure flat plane demonstrates flow characteristics analogous to those observed in air thrust bearings, in which the flow can be divided into two distinct regimes: an inertia-dominated regime and a viscosity-dominated regime. In contrast, for the pure arc plane, only an inertia-driven acceleration regime is observed.

However, the streamline analysis also reveals flow features that have been largely overlooked in previous studies of the nozzle–cylindrical flapper configuration. As the air flows through the clearance between the nozzle and the cylindrical flapper, significant recirculation is induced due to the wrapping and turning of the flow. This recirculation phenomenon is non-negligible and indicates that certain assumptions adopted in earlier flow models require further correction.

**Figure 10:**
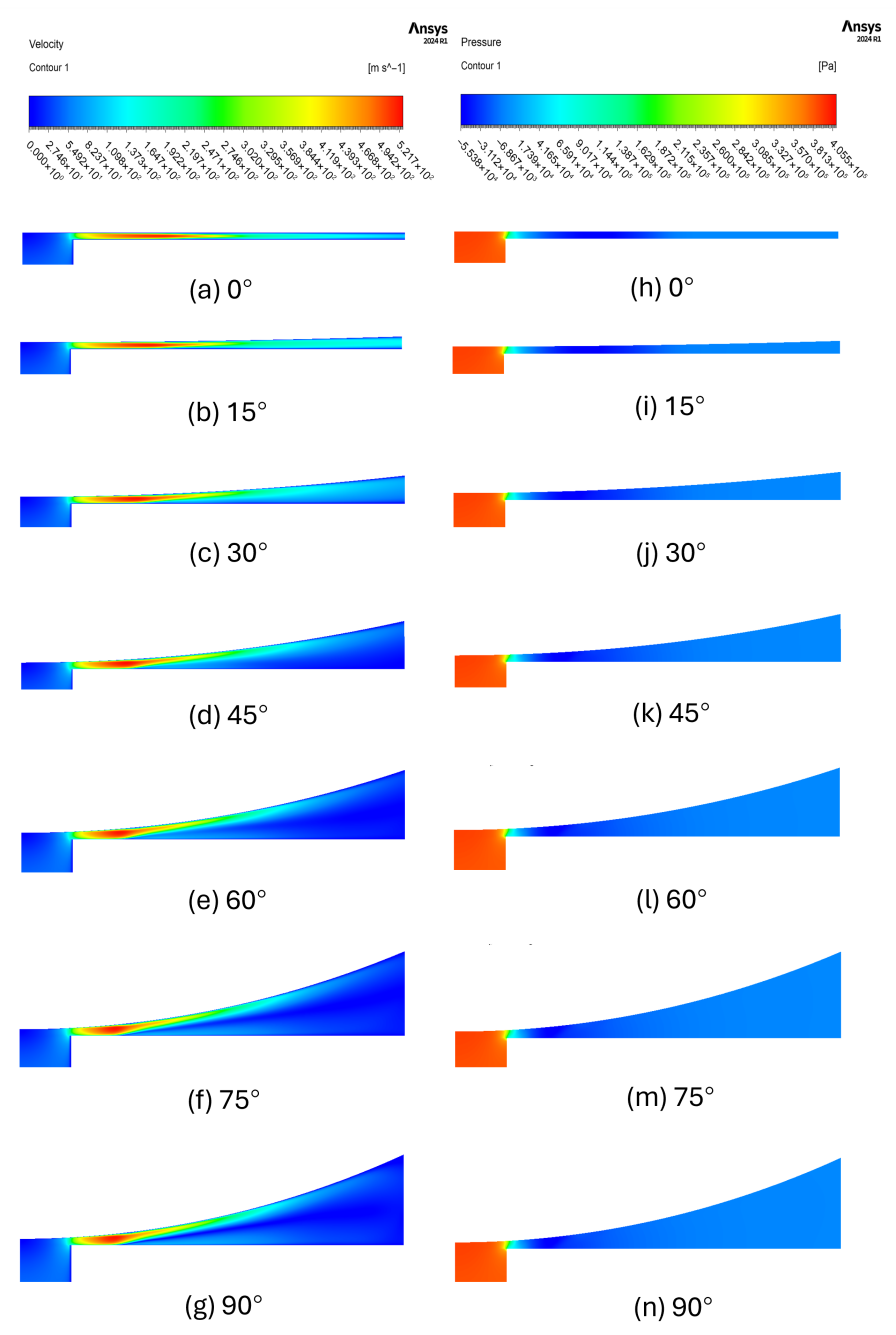
The effect of curvature variation on the flow state between the nozzle and the cylindrical flapper. (**a**–**g**) show the velocity distributions on each plane, while (**h**–**n**) present the corresponding pressure distributions. The planes are oriented at angles to the axis of the cylindrical flapper increasing from $0^{\circ }$ to $90^{\circ }$, from top to bottom.

First, the planar flow characteristics of the 45 μm clearance case are analyzed for planes oriented at angles ranging from $0^{\circ }$ to $90^{\circ }$ with respect to the axis of the cylindrical flapper. As shown in Figure 10, velocity and pressure contour plots are extracted on each plane for the 45 μm case. Panels (a–g) present the velocity distributions for planes with angles increasing from $0^{\circ }$ to $90^{\circ }$ from top to bottom, while panels (h–n) show the corresponding pressure distributions.

By comparing the velocity contours on different planes, it can be observed that when the angle is $0^{\circ }$, corresponding to the pure flat plane, the discharge behavior is fully consistent with that of an air thrust bearing. When the clearance distance along the nozzle–cylindrical flapper direction remains constant, the flow undergoes a transition from an inertia-dominated regime to a viscosity-dominated regime. This transition is reflected in the velocity evolution, which changes from a relatively uniform cross-sectional velocity distribution with gradual acceleration along the flow direction to a subsequent deceleration stage associated with the progressive development of the boundary layer.

To more clearly illustrate the flow characteristics of the pure flat plane discussed above, the streamwise velocity distributions along the clearance between the nozzle and the cylindrical flapper are extracted for the $0^{\circ }$ plane of the 45 μm clearance case. Velocity profiles across the clearance are sampled at streamwise distances of 0, 0.3, 0.6, 0.9, 1.2, 1.5, 1.8, 2.1, and 2.4 mm measured from the edge of the nozzle orifice.

**Figure 11:**
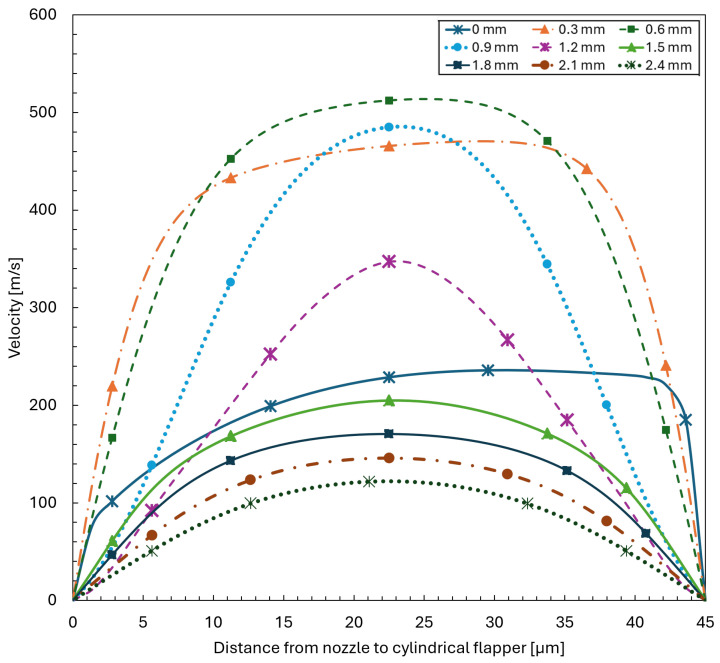
Velocity distributions across the clearance along the streamwise direction from 0 to 2.4 mm measured from the nozzle orifice on the $0^{\circ }$ plane for a nozzle–cylindrical flapper clearance of 45 μm.

As shown in Figure 11, the data at 0 and 0.3 mm indicate that the flow enters an inertia-dominated regime. In this region, the velocity distribution across the clearance is relatively uniform and exhibits a clear accelerating tendency. In contrast, within the range from 0.6 to 2.4 mm, the flow velocity gradually decreases and a boundary-layer-type velocity profile progressively develops. This behavior is consistent with the characteristics of a viscosity-dominated regime.

On the other hand, Figure 10 also indicates that for planes oriented from $15^{\circ }$ to $90^{\circ }$, the curvature of the plane increases progressively. As a result, the effective clearance through which the air flows along the streamwise direction gradually enlarges, leading to the gradual disappearance of the viscosity-dominated regime. Consequently, the flow increasingly exhibits only an inertia-dominated acceleration regime. This behavior can be attributed to the increase in the streamwise clearance distance, which prevents the airflow from fully adhering to the wall surface and thus inhibits the proper development of a boundary layer.

**Figure 12:**
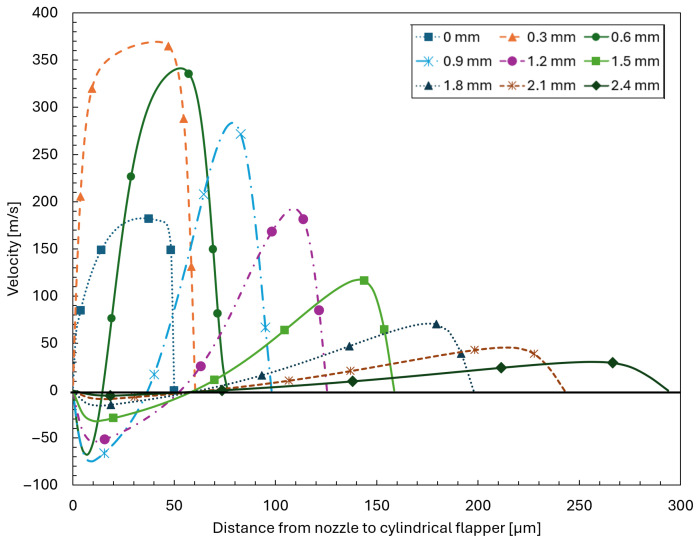
Velocity distributions across the clearance along the streamwise direction from 0 to 2.4 mm measured from the nozzle orifice on the $45^{\circ }$ plane for a nozzle–cylindrical flapper clearance of 45 μm.

**Figure 13:**
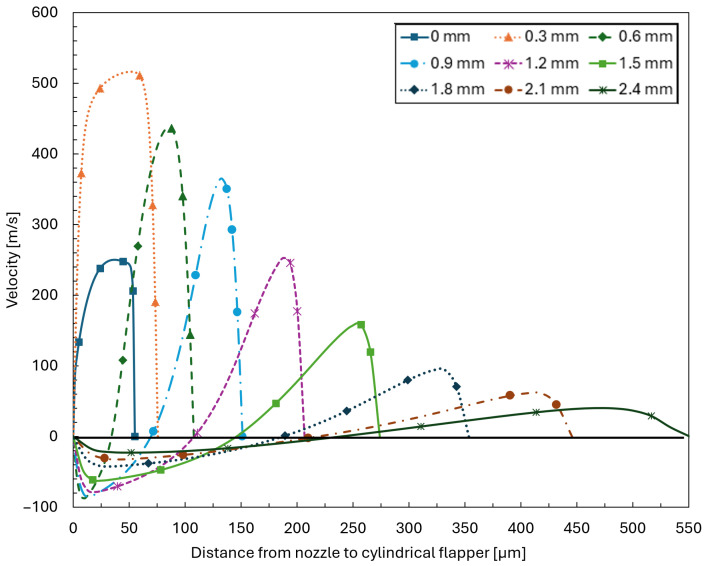
Velocity distributions across the clearance along the streamwise direction from 0 to 2.4 mm measured from the nozzle orifice on the $90^{\circ }$ plane for a nozzle–cylindrical flapper clearance of 45 μm.

Conversely, due to the wrapping and turning of the flow around the cylindrical flapper, an additional effect emerges. As the angle between the plane and the axis of the cylindrical flapper increases, corresponding to higher curvature, the airflow progressively induces recirculation near the nozzle wall. This recirculation effect becomes more pronounced with increasing plane angle. The above observations can be clearly confirmed by the streamline patterns shown in Figure 12 for the $45^{\circ }$ plane and Figure 13 for the $90^{\circ }$ plane. From a physical perspective, as the angle between the plane and the cylindrical flapper increases, leading to higher curvature, the enlargement of the effective clearance along the streamwise direction enhances the inertia-driven acceleration of the flow. At the same time, the increased curvature intensifies flow turning and wrapping around the cylindrical surface, which promotes flow separation near the nozzle wall and consequently strengthens the recirculation effect. These two effects therefore coexist and become more pronounced with increasing plane angle.

**Figure 14:**
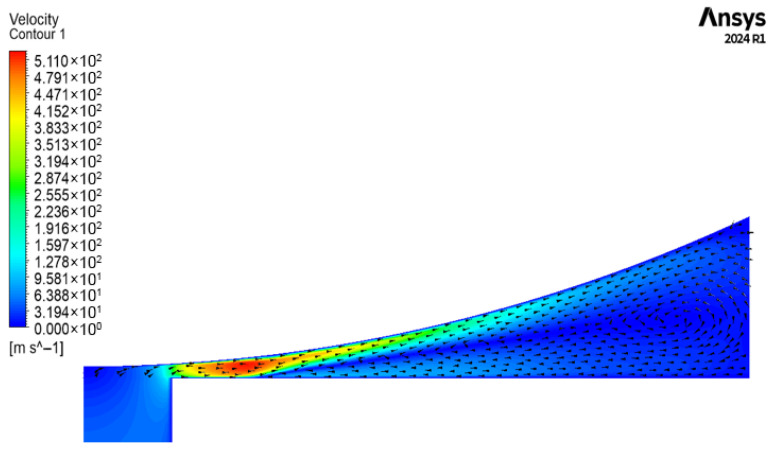
Flow pattern of the airflow on the $90^{\circ }$ plane for the 45 μm clearance case.

To more clearly visualize the influence of the recirculation effect, velocity contour and vector plots are presented for the $90^{\circ }$ plane of the 45 μm clearance case, as shown in Figure 14. It can be observed that when the curvature is sufficiently large, the recirculation region occupies a substantial portion of the clearance space between the nozzle and the cylindrical flapper. Combined with the observations in Figure 13, it becomes evident that, for planes with curvature, the evaluation of mass flow rate is significantly complicated by the presence of recirculation, particularly at streamwise locations farther away from the nozzle hole.

As a consequence, the mass flow rate calculated on curved planes may suffer from increased uncertainty if the evaluation is performed at downstream locations. To mitigate this source of error, the present study proposes evaluating the planar mass flow rate directly from the velocity and density distributions extracted at the location where the streamwise distance from the nozzle orifice is zero. This approach effectively avoids the influence of recirculation and provides a more robust basis for mass flow rate estimation.

**Figure 15:**
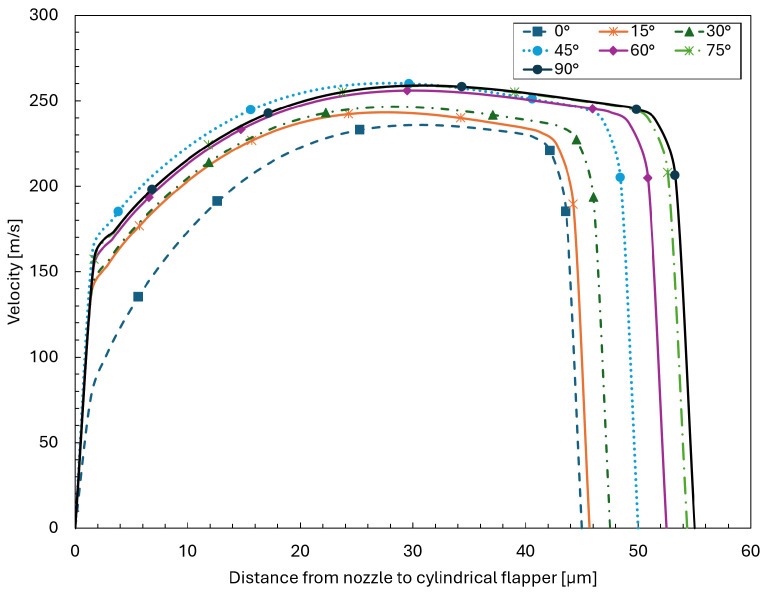
Velocity distributions across the clearance direction at a streamwise distance of zero from the nozzle orifice for planes oriented from $0^{\circ }$ to $90^{\circ }$ in the 45 μm clearance case.

**Figure 16:**
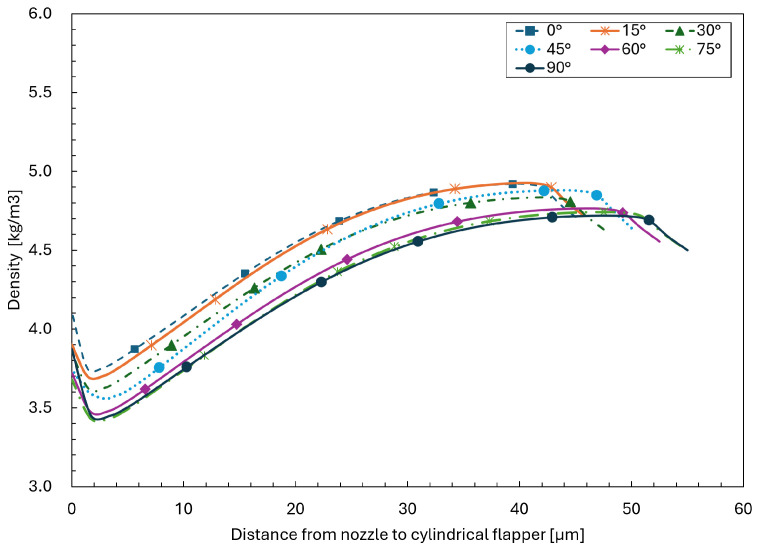
Density distributions across the clearance direction at a streamwise distance of zero from the nozzle orifice for planes oriented from $0^{\circ }$ to $90^{\circ }$ in the 45 μm clearance case.

As shown in Figure 15 and Figure 16, the velocity and density distributions across the clearance direction at a streamwise distance of zero from the nozzle orifice are extracted for planes oriented from $0^{\circ }$ to $90^{\circ }$ in the 45 μm clearance case. It can be observed that, at this location, even for the $90^{\circ }$ plane corresponding to the pure arc plane, the velocity distribution still exhibits the presence of recirculation.

Therefore, by combining the velocity distributions at this location with the corresponding density distributions across the clearance direction and the effective cross-sectional area, the mass flow rates for planes with different angles can be evaluated.

**Figure 17:**
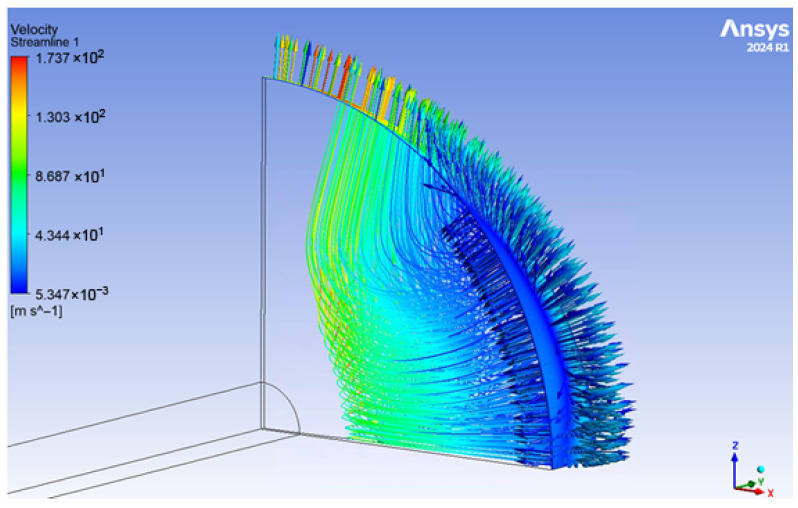
Velocity contours and vector field distributions at the outlet in the 45 μm clearance case.

To further investigate the influence of recirculation, the following analysis is conducted. The backflow area is $5.77\times 10^{-7}\;m^{2}$, while the total cross-sectional area is $1.57\times 10^{-6}\;m^{2}$, corresponding to a backflow area fraction of approximately 36.7%. The momentum flux associated with the backflow region is $6.74\times 10^{-5}\;N$, whereas the discharge momentum flux is $2.42\times 10^{-3}\;N$ and the total outlet momentum flux is $2.49\times 10^{-3}\;N$. These results indicate that, although the backflow occupies a noticeable area fraction, its contribution to the overall momentum is relatively small. This observation is further supported by the vorticity magnitude, which is approximately $9.69\times 10^{4}\;s^{-1}$ in the backflow region, compared to $6.50\times 10^{5}\;s^{-1}$ in the discharge region, indicating that the rotational intensity of the recirculating flow is significantly weaker than that of the main flow. Therefore, the influence of the backflow on the nozzle–cylindrical flapper interaction is limited. Furthermore, this conclusion is consistent with the velocity contours and vector field distributions at the outlet, which show that the main flow remains dominant despite the presence of recirculation, as shown in Figure 17.

**Figure 18:**
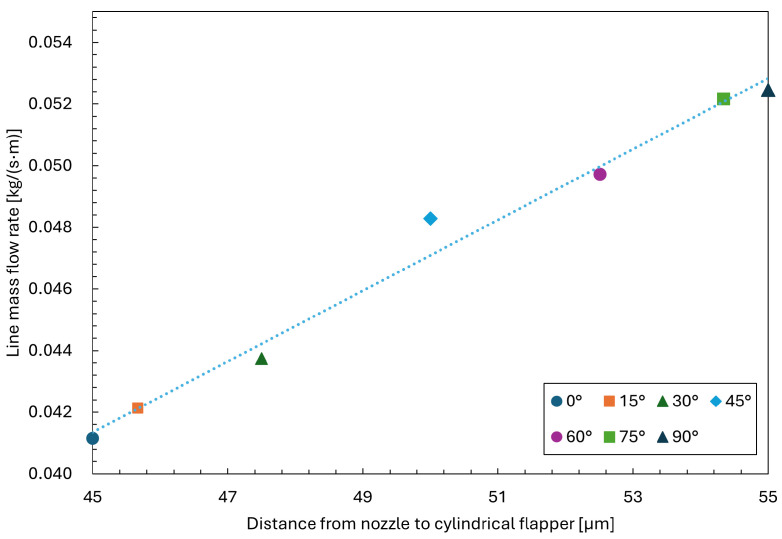
Relationship between the nozzle–flapper distance and the line mass flow rate on planes with different angles.

Based on the procedure described above, the mass flow rates on planes with different angles are evaluated. As shown in Figure 18, the relationship between the nozzle–flapper distance and the corresponding line mass flow rate is presented for each angular plane at a streamwise distance of zero from the nozzle orifice. The results indicate that the plane oriented at $90^{\circ }$, which has the largest angle relative to the axis of the cylindrical flapper and thus the highest curvature, exhibits the maximum line mass flow rate.

Moreover, the figure reveals a positive correlation between plane curvature and line mass flow rate. These observations further validate the conclusions discussed previously, demonstrating that the proposed interpretation remains reasonable and quantitatively reliable even under the additional influence of recirculation effects. From a physical perspective, under the microscale and high-velocity conditions of the nozzle–cylindrical flapper configuration, the jet exhibits a clear tendency to attach to the curved flapper surface due to the Coandă effect, while simultaneously separating from the nozzle-side plane, leading to the formation of localized recirculation regions. However, compared with macroscale flows, the influence of such recirculation on the overall high-speed microscale flow field is relatively limited, primarily due to the dominance of viscous effects and the confined geometric scale.

**Figure 19:**
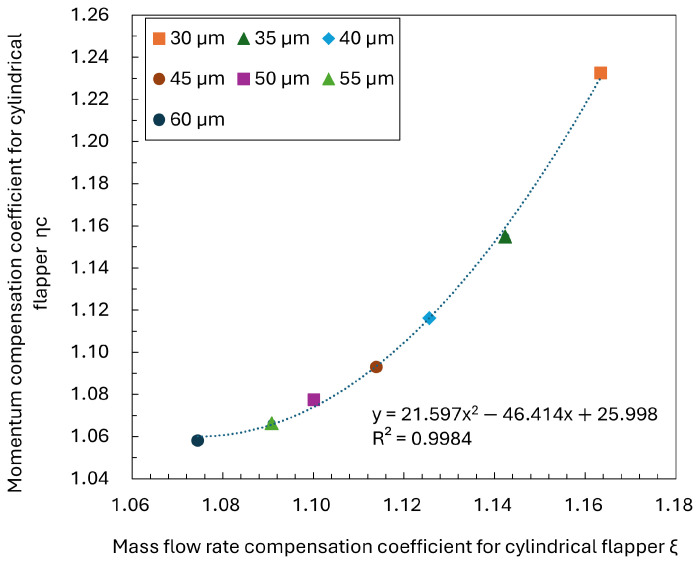
Relationship between the mass flow rate compensation coefficient and the momentum compensation coefficient for the cylindrical flapper.

As shown in Figure 19, the relationship between the mass flow rate compensation coefficient and the momentum compensation coefficient for the cylindrical flapper is presented over a nozzle–flapper clearance range of 30–60 μm. The results indicate that both coefficients increase monotonically with increasing clearance. Furthermore, a clear positive correlation ξ between plane curvature and line mass flow rate is observed, which exhibits a pronounced quadratic trend.

This behavior can be theoretically interpreted based on the formulation of the momentum coefficient. The momentum coefficient can be expressed as

(6) $$\eta_{c}=\xi \;\cdot \;C_{v}$$

where $C_{v}$ is the velocity coefficient. According to the continuity equation, the velocity coefficient can be approximated as

(7) $$C_{v}\propto \frac{\xi }{C_{c}}$$

where $C_{c}$ is the contraction coefficient. Therefore, the momentum coefficient scales is

(8) $$\eta_{c}\propto \frac{\xi^{2}}{C_{c}}$$

indicating that the quadratic relationship is a general and expected trend. These findings further corroborate the previous discussions, demonstrating that the proposed interpretation remains valid and quantitatively reliable even in the presence of recirculation effects.

Based on the results obtained in Figure 19 and following the calculation formula [8] for the mass fraction β established in previous studies, the β value of 47.2% is a calculated value derived from the numerical results obtained in the present study. This value is in close agreement with the experimentally obtained β value of 46.5%, which was calculated under identical operating conditions, namely an orifice diameter of 0.4 mm, a nozzle diameter of 0.8 mm, and a supply pressure of 500 kPaG.

**Figure 20:**
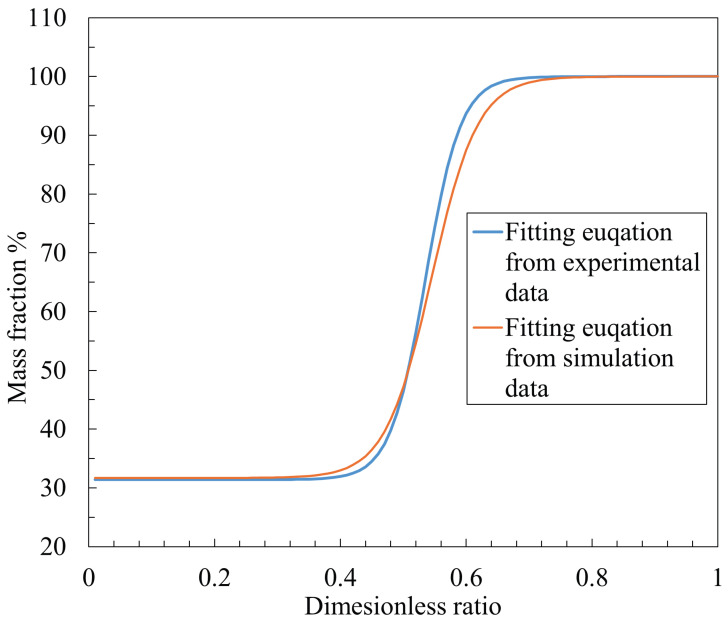
Comparison of fitted functions obtained from simulations and experiments for the ϕ–$\beta_{l}$ relationship.

The small discrepancy between the two values is attributed to the low-velocity backflow effect. Although the backflow effect can have a relatively significant influence in cases with small clearances, such as 30 μm, the β value is defined as an averaged quantity over a clearance range. Consequently, when evaluating the overall β value over the clearance interval from 30 to 60 μm, the influence of the backflow effect is effectively averaged out, resulting in a reduced impact on the final value. To further validate the feasibility of the proposed methodology, additional simulation cases were conducted under the same boundary conditions. Specifically, configurations with a nozzle diameter of 1.2mm and an orifice diameter of 0.5mm (which dimensionless ϕ = 0.42), as well as a nozzle diameter of 0.8mm and an orifice diameter of 0.5mm (which dimensionless ϕ = 0.63), were considered, with gap sizes ranging from 30 to 60μm. Using the same post-processing procedure, the obtained β values from the simulations are 93.8% and 33.9%, respectively, while the corresponding experimental values are 97.7% and 32.5%. A sigmoid fitting approach, consistent with Ref. [8], was applied to derive the fitting equations for both simulation and experimental data. As shown in Figure 20, the fitting curve obtained from the simulation results exhibits good agreement with that derived from the experimental data, demonstrating the reliability and effectiveness of the proposed numerical approach. Therefore, the CFD simulations are able to provide an accurate estimation of the β value.

From the above conclusions, two key points can be drawn. First, the static characteristics of a nozzle–cylindrical flapper system can be accurately obtained through CFD simulations. Second, the flow assumptions adopted in previous studies are shown to be physically reasonable and numerically consistent.

The flow behavior observed in the present nozzle–cylindrical flapper system shows clear similarities to classical jet impingement on convex surfaces. In classical studies, it has been reported that when a jet interacts with a convex surface, the flow tends to attach to the surface due to pressure gradients and entrainment effects, commonly associated with the Coandă effect. Consistent with these classical findings, the present results demonstrate that the airflow exhibits a tendency to attach to the cylindrical flapper surface, accompanied by curvature-enhanced acceleration and suppression of boundary layer development. In addition, the formation of recirculation regions near the nozzle wall is also consistent with previously reported flow separation and reattachment phenomena in convex surface impingement. However, the present configuration differs significantly from classical cases in that the flow is confined within a micron-scale clearance and strongly coupled with pressure feedback. These factors lead to distinct characteristics, including enhanced sensitivity of mass flow rate to clearance variation and a modified momentum distribution. Therefore, while the fundamental flow mechanisms remain consistent with classical fluid dynamics, the present system represents a micro-scale, pressure-coupled extension of convex surface jet impingement, providing new insights into its behavior under confined conditions.

Moreover, if the velocity modifications induced by recirculation effects can be fully characterized, it is possible to establish a purely theoretical model for the nozzle–cylindrical flapper system that is independent of experimental data.

## 5. Conclusions

In this study, a comprehensive three-dimensional CFD investigation of a pneumatic nozzle–cylindrical flapper system was conducted to clarify its flow behavior and static characteristics. Owing to the geometric non-axisymmetry and micron-scale clearance inherent in the nozzle–cylindrical flapper configuration, conventional two-dimensional modeling and experimental flow visualization techniques are insufficient to capture the actual discharge mechanisms. The proposed 3D CFD framework was therefore established and successfully validated against experimental measurements.

However, it should be noted that the experimental reference clearance is subject to inherent uncertainty due to surface machining roughness and misalignment tolerance (approximately ±27μm). As a result, the exact zero-clearance position cannot be determined with the same precision as in CFD simulations, where ideal smooth surfaces are assumed.

After defining a consistent reference point, the simulated results show good agreement with experimental data within the measurement uncertainty, confirming the reliability of the numerical approach. To enable a consistent comparison, an offset-based alignment was adopted between simulation and experiment, which may introduce minor discrepancies in the absolute pressure values. Therefore, the validation focuses primarily on the relative variation trends rather than the absolute magnitude.

Detailed flow field analysis reveals that curvature plays a dominant role in shaping the discharge behavior. For small clearances, increasing curvature enhances inertia-driven acceleration, suppresses boundary layer development, and reduces viscous resistance compared with conventional nozzle–flat flapper systems. At the same time, curvature-induced flow wrapping leads to pronounced recirculation near the nozzle lip, which affects mass flow rate evaluation and momentum balance. These competing mechanisms explain the observed positive correlation between plane curvature and line mass flow rate.

By introducing a mass flow rate compensation coefficient and correlating it with the momentum compensation coefficient obtained in previous studies, a strong quadratic relationship between the two coefficients is identified. This result confirms that both corrections originate from the same recirculation-induced velocity modification mechanism. Consequently, the physical assumptions underlying the previously proposed semi-empirical model are shown to be both reasonable and numerically consistent.

Overall, this work demonstrates that accurate static characteristics of nozzle–cylindrical flapper systems can be obtained solely through CFD simulations, significantly reducing dependence on costly experimental calibration. Moreover, if the recirculation-induced velocity modification can be further quantified, a fully theoretical nozzle–cylindrical flapper model independent of experimental data becomes feasible. The findings of this study provide a solid theoretical and numerical foundation for the design and application of nozzle–cylindrical flapper systems in high-speed precision pneumatic control.

## Figures and Tables

**Table 1:** Design parameters and operating conditions of the nozzle–cylindrical flapper system.

Parameters	Value
Orifice diameter, $d_{\mathrm{ori}}$	0.4 mm
Orifice length, $l_{\mathrm{ori}}$	1 mm
Nozzle diameter, $d_{\mathrm{noz}}$	0.8 mm
Nozzle lip length, *l*	3 mm
Nozzle length, $l_{\mathrm{noz}}$	6.5 mm
Cylindrical flapper diameter, $D_{f}$	16 mm
Control room diameter, $d_{\mathrm{ctrl}}$	3 mm
Control room length, *l*	3 mm
Nozzle length, $l_{\mathrm{ctrl}}$	8 mm
Cylindrical flapper diameter, $D_{f}$	16 mm
Clearance between nozzle and flapper, *x*	30 μm
Inlet, $P_{s}$	500 kPaG
Outlet, $P_{a}$	0 kPaG

**Table 2:** Mesh independence study for the nozzle–cylindrical flapper system.

Mesh ID	Number of Elements	Mass Flow Rate
1	52,427	$2.6672\times 10^{-5}$
2	109,928	$2.6161\times 10^{-5}$
3	384,120	$2.5665\times 10^{-5}$
4	863,146	$2.5260\times 10^{-5}$
5	1,237,353	$2.5109\times 10^{-5}$
6	1,884,844	$2.4958\times 10^{-5}$
7	2,881,402	$2.4796\times 10^{-5}$

## Data Availability

The original contributions presented in this study are included in the article. Further inquiries can be directed to the corresponding author.
